# Cerebral baylisascariosis in a rainbow lorikeet (*Trichoglossus moluccanus*) in a German Zoo

**DOI:** 10.1186/s12917-024-03946-8

**Published:** 2024-03-08

**Authors:** Sarah Pfetzing, Andreas Bernhard, Christian Bauer, Florian Hansmann

**Affiliations:** 1 Institute of Veterinary Pathology, Faculty of Veterinary Medicine, Leipzig University, An den Tierkliniken 33, 04103 Leipzig, Germany; 2 Zoo Leipzig GmbH, Pfaffendorfer Straße 29, 04105 Leipzig, Germany; 3 Institute of Parasitology, Justus Liebig University Giessen, Schubertstraße 81, 35392 Giessen, Germany

**Keywords:** Baylisascariosis, Neural larva migrans, Encephalitis, Rainbow lorikeet, Germany

## Abstract

**Background:**

The raccoon roundworm, *Baylisascaris procyonis*, can cause a meningoencephalitis as neural larva migrans which is known in avian species, including rainbow lorikeets in North America, but has not been described in Old World parrots in Germany yet.

**Case presentation:**

A 2-month-old, male rainbow lorikeet from a zoo in Germany was submitted for necropsy. Prior to death the animal had progressive neurological signs like apathy and torticollis. In the cerebrum a focally extensive severe granulomatous to necrotizing encephalitis with an intralesional larval nematode was diagnosed. Based on the clinical and pathological findings, the larval morphology and the epidemiological background, the larva was identified as *Baylisascaris procyonis*.

**Conclusions:**

Cerebral baylisascariosis should be considered as a differential diagnosis in zoo and pet birds with neurological signs having contact to racoons or rather racoon faeces in Germany due to the high prevalence of *Baylisascaris procyonis* in the German raccoon population.

## Background

*Baylisacaris procyonis* is an intestinal nematode species with raccoons (*Procyon lotor*) as definitive hosts [1]. Infected raccoons can excrete millions of non-embryonated *Baylisascaris* eggs with their feces, where the eggs embryonate and then contain the infective larva [2]. Eggs can remain infectious for many years within environment. *Baylisascaris procyonis* develops in a facultative heteroxenous life cycle: raccoons become infected by oral uptake of the infective eggs from the environment (direct life cycle) or by ingesting intermediate hosts (indirect life cycle). A wide range of animal species can act as intermediate hosts in the life cycle of this parasite, particularly rodents and birds; they usually become infected by ingesting embryonated eggs [1–3]. In intermediate hosts, the larvae hatch and penetrate the intestinal wall transmurally. Via the blood they reach the liver, lungs or the central nervous system and eyes, growing and moulting there to third-stage larvae. This can result in extensive tissue damage and visceral, ocular or neural baylisascariosis [1, 2, 4, 5]. *Baylisascaris procyonis* is also a zoonotic pathogen. Human infection may be asymptomatic or can cause ocular larva migrans symptoms or often fatal meningoencephalitis [4, 5]. In birds, the brain is most frequently affected [2]. The consequences are necrosis, eosinophilic meningoencephalitis, malacia and spongiosis [5]. Clinically, cerebral baylisascariosis in birds is characterized by disorientation, ataxia, weakness, tremor, falling, inability to stand and death [5–7]. Here we describe the first case of a cerebral baylisascariosis in a rainbow lorikeet with fatal neurological signs in Germany.

## Case presentation

The affected male rainbow lorikeet was bred in Germany and kept with other lorikeets in inside cages and an outdoor aviary of the Leipzig Zoo. The birds were fed with fruits and vegetables and grain mix in bowls. At the age of two months, the affected animal developed progressive neurological signs, including torticollis and apathy. It was also attacked by other adult loris who plucked its tail feathers. The bird died four days after the onset of clinical signs. Wild raccoons are sighted mostly at night at the zoo; trees near the aviary would enable them to climb on top.

At necropsy, except featherless areas with multifocal erosions and ulcerations on the back and wings no significant findings were detected.

**Fig. 1:**
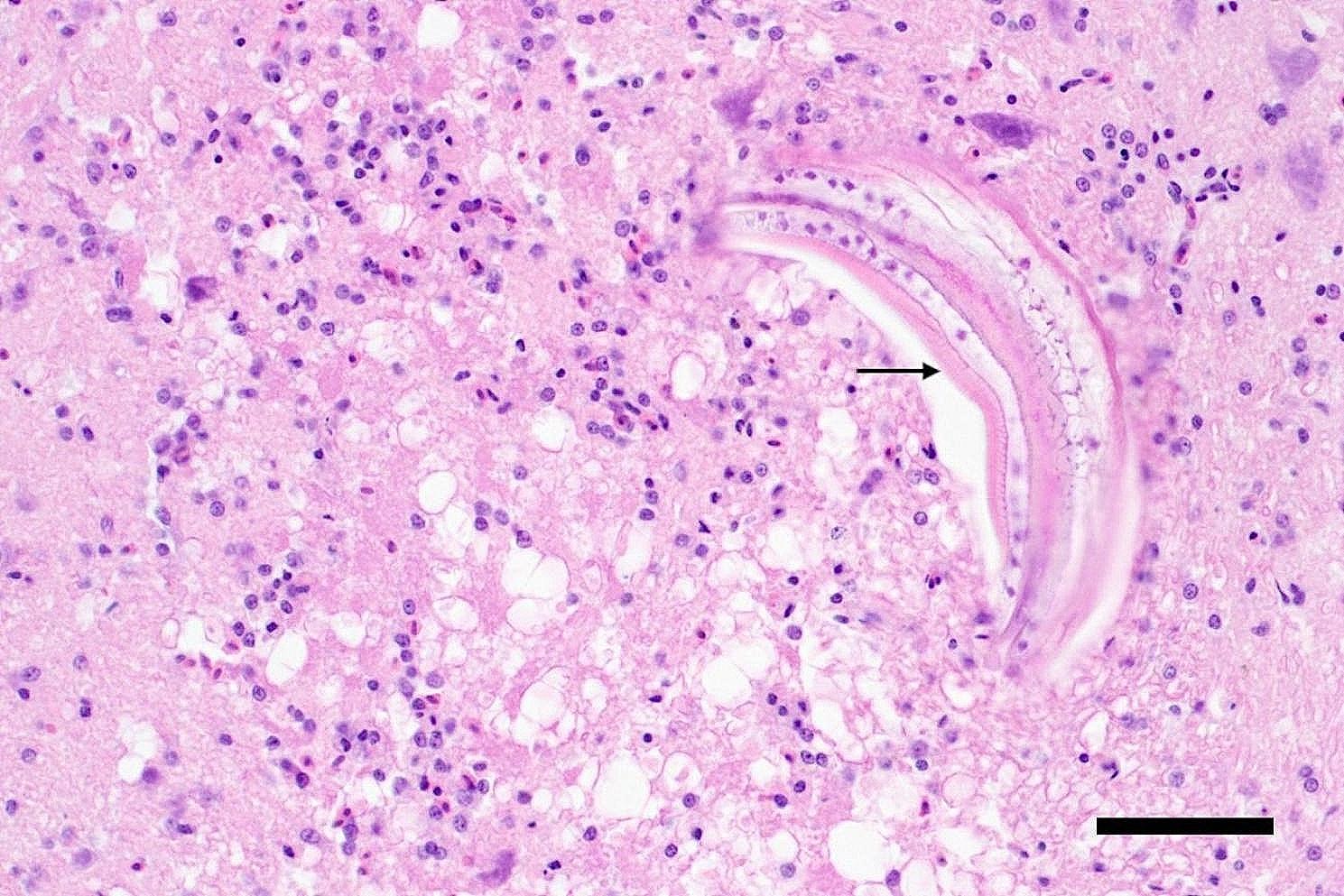
Longitudinal section of a *Baylisascaris procyonis* larva within the cerebrum of a rainbow lorikeet within the centre of a locally extensive, necrotizing and granulomatous encephalitis; cuticula (arrow), bar length 50 μm, HE

**Fig. 2:**
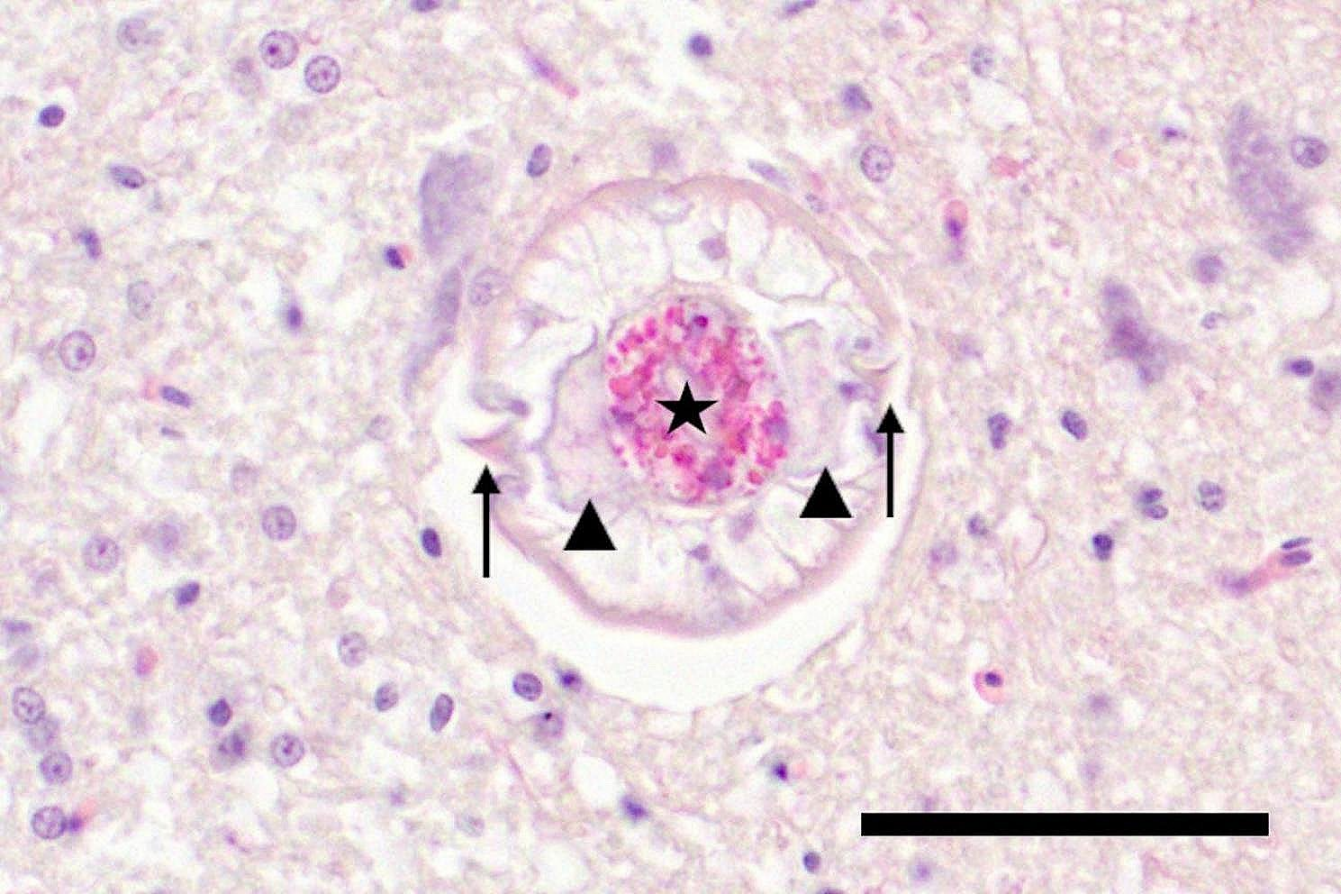
Cross section of a *Baylisascaris procyonis* larva within the cerebrum of a rainbow lorikeet showing lateral alae (arrows), the intestinal tract (asterisk) and excretory columns (arrowhead), bar length 50 μm, HE

For histopathological examination representative tissue samples from back and wing skin, pectoral muscle, heart, lung, glandular stomach, muscular stomach, intestine, liver, spleen as well as cerebrum, cerebellum, brainstem and medulla oblongata were fixed in 10% buffered formalin overnight followed by dehydration in degraded alcohols and embedded in paraffin wax. Thereafter tissue sections were stained with hematoxylin and eosin (HE). Additional histochemical staining of wing skin samples, including periodic acid–Schiff reaction (PAS reaction) and Fite-Faraco staining, was performed as described [8]. Histologically, in the cerebrum a locally extensive, moderate to severe granulomatous to necrotizing encephalitis with neuronal degeneration as well as mild microgliosis was detected (Fig. 1). Centrally located within the lesion a nematode larva with a pale eosinophilic cuticle of 5 μm thickness (Fig. 1) and a diameter of 60 μm (Fig. 2) was present. The cuticle had two lateral alae, visible as prominent, triangular, homogeneous, eosinophilic structures. Inside the larva an eosinophilic, finely punctured, oval-shaped intestinal tract and pale eosinophilic, cloud-like, triangular-shaped excretory columns were visible on both sides (Fig. 2).

An additional pathohistological finding of the wing skin included a severe, focal, ulcerative dermatitis with granulation tissue. No acid-fast bacteria or fungal structures were detected within these lesions by PAS reaction or Fite-Faraco staining.

As part of the routine diagnostic workflow PCRs for Avian Influenza A Virus and *Chlamydia psittaci* were performed and no pathogen-specific nucleic acid sequences were detected. Furthermore, examination of the gastrointestinal tract and faeces for parasite stages using a flotation-sedimentation method were negative.

## Discussion and conclusions

The intracerebral lesions and the morphology of the larva are consistent with *Baylisascaris procyonis* infection, including the presence of two bilateral alae, characteristic of ascaridoid larvae, and a diameter of 60 μm, consistent with *Baylisascaris procyonis* larvae [1, 2, 9–11]. Larvae of *Toxocara* spp. (*T. canis, T. cati*) and *Toxascaris leonina* can be excluded in the differential diagnosis due to their smaller diameter (maximum 15–37 μm) [12, 13]. The detection of *Baylisascaris procyonis* infected raccoons in the zoo’s urban environment supports this diagnosis [14]; the eggs they excreted could have served as a source of infection for the rainbow lorikeet.

*Baylisascaris procyonis* is endemic in North America and Europe; it can cause clinical baylisascariosis in numerous intermediate host species, especially birds and rodents [1, 5, 9]. In Europe this parasite is considered to be an emerging pathogen [15]. Raccoons are widespread in Germany and often live near people [14, 16]. Genetic studies show that raccoons in Leipzig, Germany and the surrounding area genetically belong to two founder populations (“Hessen” and “Harz”) with both of them being carriers of *Baylisascaris procyonis* [17]. In the raccoon population of Leipzig the prevalence of *Baylisascaris procyonis* was about 75% [14]. In other studies the prevalence in German raccoons was 28.7% respectively 43.6% [18, 19]. The high density of excreted eggs in raccoon faeces ranges from 20,000 to 26,000 *Baylisascaris procyonis* eggs / g thereby serving as significant hazard for humans and animals [2, 4]. However, it has to be taken into account that not all raccoon populations in Germany are carriers of *Baylisascaris procyonis* [17]. Furthermore, recent studies show a transmission of *Baylisascaris procyonis* in a roundworm-free raccoon population indicating that the distribution of *Bayliscaris procyonis* is not restricted to the range of raccoons [20]. Therefore, there seems to be a significant risk of infection for suitable intermediate hosts in Germany. Rainbow lorikeets in the Leipzig Zoo were kept in inside cages and an outdoor aviary. Contamination with raccoon droppings seems possible since wild raccoons have been observed in the zoo and can climb on the trees above the aviary. This was probably the source of the *Baylisascaris procyonis* infection. A risk of infection with *Baylisascaris procyonis* for other zoo animals and humans (animal caretakers and zoo visitors) must also be assumed [7].

Single cerebral *Baylisascaris procyonis* larvae have been reported to cause death in mice and small birds [5]. A single *Baylisascaris* larva was found in the brain of a dead Moluccan cockatoo (*Cacatua moluccensis*), along with a cardiac granuloma caused by larval migration [6]. In five infected macaws (*Ara macao, Ara arauna*), four had only one intracerebral single *Bylisascaris* larva, in one two larvae were detected within the cerebrum [21]. In the case of the rainbow lorikeet presented here, also only a single larva was found in the brain.

In parrots, the course of clinical baylisascariosis is short and fatal: a Patagonian parakeet (*Cyanoliseus patagnus*) [7] and an Moluccan cockatoo [6] died approximately three weeks after the onset of clinical signs. Blue and gold macaws, scarlet Macaws and hybrid macaws showed clinical signs like ataxia and torticollis and two of them died 20 days after developing neurological sings or were euthanized after 29 days, respectively [21]. Two blue-fronted amazons (*Amazona aestiva*) showed coordination difficulties and torticollis, and died four weeks later or were euthanized [11]. In the case presented here, the rainbow lorikeet died 4 days after showing clinical signs (torticollis and apathy), which was faster than described in previous reports. Whether the bird size or other factors contributed to a more rapid progression of the disease in this case remains speculative. *Baylisascaris* larvae can remain in the brain without causing histological lesions [7]. However, they are also associated with malacia and necrosis, eosinophilic meningoencephalitis, spheroids and astrogliosis [3, 4, 7]. Migratory pathways of the parasite may be visible through hemorrhage or neuroaxonal degeneration [2, 4, 6]. Further multinucleated giant cells, plasma cells, histiocytes and lymphocytes are often present [3, 4]. Comparable lesions were noted in the present case. Here, the larva of *Baylisascaris procyonis* coalesced with degenerating neurons, oedema and granulomatous encephalitis. The absence of granulomas with surrounding fibrosis indicates an acute course of infection [4]. In the present case, the young age of the animal may have contributed to the rapid course of the disease, although a direct influence of age on the disease course in cerebral baylisascariosis has not been described yet.

The skin lesions observed in the present rainbow lorikeet, consisting of focal ulcerative dermatitis with granulation tissue, were a secondary finding and most likely related to the attack of other birds within the aviary.

In conclusion, cerebral baylisascariosis should be considered as a differential diagnosis in zoo and pet birds with neurological signs having contact to racoons or rather racoon faeces in Germany due to the high prevalence of *Baylisascaris procyonis* within the raccoon population.

## Data Availability

All data generated or analysed during this study are included in the article.

## Abbreviations

HE: Hematoxylin and eosin
PAS reaction: Periodic acid–Schiff reaction
